# Incidence and risk factors of hepatitis B virus reactivation in patients with multiple myeloma in an era with novel agents: a nationwide retrospective study in Japan

**DOI:** 10.1038/s41408-017-0002-2

**Published:** 2017-11-23

**Authors:** Yutaka Tsukune, Makoto Sasaki, Takeshi Odajima, Kazutaka Sunami, Tomomi Takei, Yukiyoshi Moriuchi, Masaki Iino, Atsushi Isoda, Aya Nakaya, Tsuyoshi Muta, Takaaki Miyake, Koji Miyazaki, Takayuki Shimizu, Kei Nakajima, Aiko Igarashi, Koji Nagafuji, Taro Kurihara, Tomonori Aoyama, Hiroki Sugimori, Norio Komatsu

**Affiliations:** 1 Department of Hematology, Juntendo University School of Medicine, Bunkyo-ku, Tokyo 113-8421 Japan; 2 Faculty of Health Science, Daito Bunka University, School of Sports and Health Science, Higashi-Matsuyama, Saitama 355-8501 Japan; 3 Department of Hematology, National Hospital Organization Okayama Medical Center, Okayama, Okayama 701-1192 Japan; 4 Department of Hematology, Japanese Red Cross Medical Center, Shibuya-ku, Tokyo 150-8935 Japan; 5 Department of Hematology, Sasebo City General Hospital, Sasebo, 857-0056 Nagasaki Japan; 6 Department of Hematology, Yamanashi Prefectural Central Hospital, Kofu, Yamanashi 400-8506 Japan; 7 Department of Hematology, National Hospital Organization Shibukawa Medical Center, Shibukawa, Gunma 377-0280 Japan; 8 First Department of Internal Medicine, Kansai Medical University, Hirakata, Osaka 573-1010 Japan; 9 Department of Hematology/Oncology, Japan Community Health Care Organization Kyushu Hospital, Kita-Kyusyu, Fukuoka 806-8501 Japan; 10 Department of Oncology/Hematology, Shimane University Hospital, Izumo, Shimane 693-8501 Japan; 11 Department of Transfusion and Cell Transplantation, Kitasato University School of Medicine, Sagamihara, Kanagawa 252-0374 Japan; 12 Division of Hematology, Department of Medicine, Keio University School of Medicine, Shinjuku-ku, Tokyo 160-8582 Japan; 13 Department of Hematology/Oncology, University of Yamanashi, Chuo, Yamanashi 409-3898 Japan; 14 Hematology Division, Tokyo Metropolitan Cancer and Infectious Diseases Center, Komagome Hospital, Bukyo-ku, Tokyo 113-8677 Japan; 15 Division of Hematology and Oncology, Department of Medicine, Kurume University School of Medicine, Kurume, Fukuoka 830-0011 Japan; 16 Department of Internal Medicine, Niigata Cancer Center Hospital, Niigata, Niigata 951-8566 Japan; 17 Department of Gastroenterology, Juntendo University School of Medicine, Bunkyo-ku, Tokyo 113-8421 Japan; 18 Department of Preventive Medicine, Daito Bunka University, Graduate School of Sports and Health Science, Higashi-Matsuyama, Saitama 355-8501 Japan

An estimated two billion people worldwide have been infected with the hepatitis B virus (HBV). Specifically, the prevalence of HBV infection is particularly high in Asia, including Japan. HBV reactivation (HBVr) can occur in HBV carriers and in patients with resolved HBV infection who are receiving cancer chemotherapy. HBVr can induce severe flares of hepatitis and lead to fatal fulminant hepatitis. With the increasing availability of rituximab-based regimens, HBVr has become a well-known complication of lymphoma chemotherapy. However, before the era of novel agents, such as proteasome inhibitors and immunomodulatory drugs (IMiDs), there were few reports of HBVr in patients with multiple myeloma (MM). Since the approval of these novel agents, the number of reports has increased^1–3^. In our previous study of 641 patients with MM, we reported that 9 of 99 (9.1%) patients with resolved HBV infection experienced HBVr^4^. Furthermore, the cumulative incidences of HBVr at 2 and 5 years were 8% and 14%, respectively. While our previous study concluded that HBVr was not rare, reasonable risk factors were not identified^4^. Therefore, this nationwide retrospective study aimed to evaluate the actual incidence and risk factors of HBVr in Japanese patients with MM.

This study included patients who were diagnosed with symptomatic MM using the International Myeloma Working Group diagnostic criteria between January 2006 and February 2016 at board certified institutes of the Japanese Society of Hematology. The terminology and definitions used remain the same as in our previous report^4^. This study was performed in accordance with the ethical principles of the Declaration of Helsinki and was approved by the ethics review board of each participating institution. Informed consent was obtained from all patients.

A multivariate logistic regression model was applied to identify independent risk factors related to HBVr using SAS statistical analysis software (version 9.4.; SAS Institute Inc., Cary, NC, USA). The other analyses were performed using the same methods as our previous report^4^.

This study collected data from 5078 patients, including data on 641 patients evaluated in our previous study, from 76 Japanese hospitals^4^. All patients had been treated with either novel agents (bortezomib, thalidomide, lenalidomide, pomalidomide, panobinostat, carfilzomib elotuzumab, and ixazomib) or had undergone autologous stem cell transplantation (auto-SCT). Of these patients, 52 (1.0%) were HBV carriers, and 760 (15.0%) exhibited resolved HBV infection. Prophylactic antiviral agents were administered to 46 (88.5%) of the 52 HBV carriers to prevent hepatitis; one of the remaining 6 developed hepatitis.

**Fig. 1:**
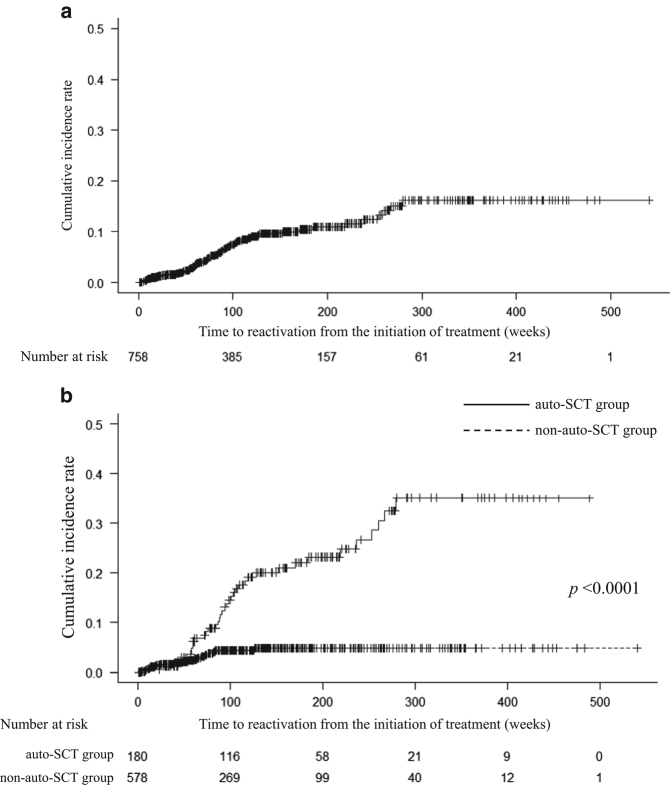
Cumulative incidences of HBV reactivation **a**, and with or without auto-SCT **b** HBV reactivation occurred in 7.6% (58/760) of all patients with resolved HBV infection. The cumulative incidences of HBV reactivation at 2 years and 5 years were 7.9% and 14.1%, respectively **a**. HBV reactivation occurred in 21.1% (38/180) of patients who received auto-SCT treatment and 3.4% (20/580) of patients who received novel agent treatment. The cumulative incidences at 2 years and 5 years in the auto-SCT group were 16% and 30.6%, respectively. The cumulative incidences at 2 and 5 years in the novel agents group were 4.4% and 4.8%, respectively. The incidence rate was significantly higher in the auto-SCT group than in the novel agents group (*p* < 0.0001) **b**

Baseline characteristics of MM patients with resolved HBV infection are shown in Table 1. We identified 180 (23.7%) patients who underwent auto-SCT (156 cases: upfront single, 13 cases: upfront tandem, 8 cases: relapse, 3 cases: upfront and relapse), and 178 patients received high-dose melphalan with or without bortezomib as a conditioning regimen. Sixty-one patients received post-auto-SCT maintenance therapy. During a median follow-up period of 101 weeks (range: 1–541 weeks), 58 of 758 (7.7%) patients with resolved HBV infection experienced HBVr. The cumulative incidence rates of HBVr at 2 and 5 years were 7.9% and 14.1%, respectively (Fig. 1a). Ten of fifty-eight (17.2%) patients with HBVr developed hepatitis, and one died of fulminant hepatitis despite the administration of antiviral agent. In these 10 patients, HBVr was diagnosed after an elevation of alanine aminotransferase levels was observed. Conversely, the other patients who had regular monitoring of HBV-DNA and/or preemptive antiviral therapy according to the Japan Society of Hepatology guidelines did not develop hepatitis^5^.

**Table 1:** Baseline characteristics of patients with resolved hepatitis B virus infection

	HBV reactivation (*n* = 58)	No HBV reactivation (*n* = 702)	*p*-Value
Age			<0.0001
Mean (range)	64 (44–83)	70 (43–93)	
Male, *n* (%)	34 (58.6)	397 (56.6)	0.7600
MM subtype, *n* (%)			0.1769
IgG	30 (51.7)	376 (53.6)	
IgA	12 (20.7)	181 (25.8)	
IgD	4 (6.9)	15 (2.1)	
Light chain only	12 (20.7)	115 (16.4)	
Others^a^	0 (0.0)	15 (2.1)	
Durie–Salmon staging system, *n* (%)			0.9108
IA	4 (6.9)	58 (8.3)	
IB	0 (0.0)	6 (0.9)	
IIA	16 (27.6)	164 (23.4)	
IIB	1 (1.7)	26 (3.7)	
IIIA	29 (50.0)	336 (47.9)	
IIIB	7 (12.1)	104 (14.8)	
Unknown	1 (1.7)	8 (1.1)	
International staging system, *n* (%)			0.0943
I	19 (32.8)	131 (18.7)	
II	21 (36.2)	297 (42.3)	
III	16 (27.6)	267 (38.0)	
Unknown	2 (3.4)	7 (1.0)	
HBV serological marker, n (%)			0.1447
Anti-HBs negative	17 (29.3)	163 (23.2)	
WBC (×10% μL)			0.4892
Mean (range)	5.2 (2.1–9.3)	5.3 (1.3–52.9)	
Lymphocyte (×10% μL)			0.3978
Mean (range)	1.6 (0.3–3.1)	1.5 (0.1–13.9)	
Albumin (g/dL)			0.0250
Mean (range)	3.6 (2.1–5.0)	3.4 (1.0–5.3)	
ALT (IU/L)			0.2574
Mean (range)	23 (7–73)	20 (2–160)	
Gamma-GTP (IU/L)			0.0035
Mean (range)	31 (9–115)	43 (6–540)	
Novel agent, *n* (%)			
Bortezomib	48 (82.8)	613 (87.3)	0.3210
Lenalidomide	22 (37.9)	401 (57.1)	0.0047
Thalidomide	7 (12.1)	138 (19.7)	0.1574
Steroid, *n* (%)			
Dexamethasone	53 (91.4)	648 (92.3)	0.7973
Prednisolone	14 (24.1)	259 (36.9)	0.0516
Stem cell transplantation, *n* (%)			
Auto-SCT	38 (65.5)	142 (20.2)	<0.0001
Maintenance therapy^b^	12(31.6)	49 (34.5)	0.7913
Allo-SCT	1 (1.7)	2 (0.3)	0.2122
Follow up (week)	*n* = 58	*n* = 700	—
Median (range)	74 (4–280)	105 (1–541)	

Among the 5078 patients with MM, 760 patients exhibited resolved HBV infection. Of these 760 patients, 58 patients experienced HBV reactivation. Univariate analysis revealed that age, elevated serum albumin levels, and auto-SCT treatment were significant risk factors of HBV reactivation. Conversely, the administration of lenalidomide was significantly associated with a lower prevalence of HBV reactivation

*Anti-HBs* antibodies against hepatitis B surface antigen, *WBC* white blood cells, *ALT* alanine aminotransferase, *gamma-GTP* gamma-glutamyl transpeptidase, *auto-SCT* autologous stem cell transplantation, *allo-SCT* allogeneic stem cell transplantation

^a^Others, includes IgM, non-secretary, and biclonal gammopathy (IgG and IgA)

^b^Maintenance therapy was compared in patients who received auto-SCT

In the univariate analysis, a high incidence of HBVr was observed in groups with elevated serum albumin levels, of younger age, with decreased gamma-glutamyl transpeptidase levels, and who received auto-SCT (Table 1). Multivariate analysis revealed that auto-SCT was a powerful risk factor for HBVr (adjusted odds ratio (OR) 11.56, 95% confidence interval (CI): 4.61–29.0) (Table 2). The cumulative incidence of HBVr in patients treated with auto-SCT at 2 and 5 years was significantly higher (16% vs. 30.6%, respectively) than those not treated with auto-SCT (4.4% vs. 4.8%, respectively) (log-rank test, *p* < 0.0001) (Fig. 1b). Contrastively, lenalidomide treatment was associated with a low HBVr prevalence (adjusted OR 0.47 (95% CI: 0.26–0.83)) (Table 2).

**Table 2:** Multivariate analysis of risk factors associated with hepatitis B virus reactivation

	HBV reactivation (%) (*n* = 58)		No HBV reactivation (%) (*n* = 702)		Total (%) (*n* = 760)		Unadjusted OR (95% CI)		Adjusted OR^a^ (95% CI)	
Age (years old)										
<70	40	(11.0)	324	(89.0)	364	(47.9)	2.59	(1.458–4.611)	0.52	(0.200–1.341)
≥70	18	(4.5)	378	(95.5)	396	(52.1)	1		1	
Albumin (g/dL)										
<3.4	39	(9.4)	374	(90.6)	413	(54.3)	1		1	
≥3.4	19	(5.5)	328	(94.5)	347	(45.7)	1.80	(1.020–3.177)	1.50	(0.820–2.739)
Gamma-GTP (IU/mL)										
<25	29	(8.7)	304	(91.3)	333	(43.8)	1.31	(0.766–2.238)	1.52	(0.858–2.693)
≥25	29	(6.8)	398	(93.2)	427	(56.2)	1		1	
Auto-SCT										
Done	38	(21.1)	142	(78.9)	180	(23.7)	7.49	(4.229–13.275)	11.56	(4.606–28.994)
Not done	20	(3.4)	560	(96.6)	580	(76.3)	1		1	
Lenalidomide										
Received	22	(5.2)	401	(94.8)	423	(55.7)	0.46	(0.264–0.796)	0.47	(0.261–0.830)
Not received	36	(10.7)	301	(89.3)	337	(44.3)	1		1	

Multivariate analysis revealed that only auto-SCT was independently associated with a high prevalence of HBV reactivation. In contrast, lenalidomide significantly decreased the incidence of HBV reactivation

^a^The adjusted OR was calculated by a conditional logistic regression, which was adjusted for age, albumin levels, gamma-GTP levels, auto-SCT treatment, and lenalidomide treatment

*OR* odds ratio, *CI* confidence interval, *gamma-GTP* gamma-glutamyl transpeptidase, *auto-SCT* autologous stem cell transplantation

There have only been a few reports of HBVr among MM patients who were treated with lenalidomide or pomalidomide^6^, but this adverse effect is mentioned in the drug information sheets of both drugs in the EU. In this study, only one patient did not receive auto-SCT or novel agents (except lenalidomide). Lenalidomide is an IMiD that targets cereblon (CRBN) to induce antitumor activity. The argonaute2 (AGO2) is the only member with catalytic activity and plays an essential role within the RNA-induced silencing complex; thus, it regulates small RNA-guided gene splicing processes^7^. Recently, Xu et al.^8^ showed that AGO2 was a CRBN binding partner and was negatively regulated by CRBN in MM cells. In the study, administration of lenalidomide significantly increased expression of CRBN and decreased the levels of AGO2 and microRNAs in MM cell lines. Another study reported that the knock down of AGO2 induced decreased levels of HBsAg and HBV-DNA in cells transfected with plasmids of HBV components^9^. These results suggest that lenalidomide may decrease AGO2 levels and inhibit HBV proliferation in patients with MM.

In our previous study, auto-SCT was not significantly associated with HBVr^4^; however, in the present study, auto-SCT was shown to be a powerful risk factor for HBVr. This discrepancy could be a result of different samples sizes. Auto-SCT is a well-known risk factor for HBVr^10^, and a previous Korean retrospective study that analyzed 230 patients with resolved HBV infection similarly reported that auto-SCT significantly increased the prevalence of HBVr in patients with MM^1^. Conversely, only 17 patients with MM who experienced HBVr were reported from non-Asian regions, 14 (82.4%) of these received auto-SCT. It is possible that auto-SCT is a risk factor for HBVr not only in Asia but also worldwide.

Recently, several guidelines recommend antiviral prophylaxis for patients at high risk of HBVr from the initiation of chemotherapy^11, 12^. The present study and a prospective study on lymphoma showed that patients who received preemptive antivirals following HBV-DNA monitoring did not experience HBVr-related hepatitis^13^. Additionally, HBV-DNA monitoring is more economical than antiviral prophylaxis; these results suggest that prophylactic antiviral therapy might not always be recommended in MM.

There are three differences in results between the present study and the previously described Korean study^1^. First, the cumulative incidence of HBVr in the auto-SCT group was higher in the current study (16 and 7% at 2 years), which could be attributable to the different definitions of HBVr used. In the Korean study, HBVr was defined as the reappearance of HBsAg in the blood; therefore, our definition may lead to earlier and higher incidences of HBVr. The second difference is that the current study observed a longer time between auto-SCT and HBVr^1^. In the Korean study, all patients who underwent auto-SCT experienced HBVr within 6 months, and the investigators recommended monitoring HBV-DNA levels for at least 24 months after transplantation. In the present study, 6 of the 38 patients who received auto-SCT experienced HBVr after more than 2 years post transplantation (median, 55 weeks; range, 10–250 weeks). Two of these patients were not treated with chemotherapeutic agents after auto-SCT until they exhibited HBVr (maximum, 226 weeks). These results suggest that the appropriate period of HBV-DNA monitoring for patients with MM remains unclear; long-term monitoring may be required to prevent flares of hepatitis, especially among patients treated with auto-SCT. The third difference is that the Korean study, along with several other studies, identified negative or low titers of antibodies against the hepatitis B surface antigen (anti-HBs) to be a risk factor of HBVr^1, 14^. However, there was no significant association between anti-HBs negativity and HBVr in the present study, and serological markers could not be thoroughly assessed because each institution used different assay methods.

Our study had two major limitations. First, the duration of observation was relatively short (approximately 2 years). The therapeutic outcome MM treatment using novel agents has seen significant improvements. The current systematic chemotherapy, including the consolidation and/or maintenance phase^15^, requires a longer treatment period. There was no significant association between post-transplant maintenance therapy and HBVr in our study (*p* = 0.7913). However, the actual prevalence of HBVr in MM patients who experienced prolonged treatment could not be determined, as the incidence of HBVr gradually increased up to 5 years (260 weeks) after initiating treatment. Secondly, the relationship between HBVr and allogeneic SCT or recently approved novel agents apart from bortezomib, thalidomide, and lenalidomide, was unclear. The sample size was too small to evaluate the relationship.

In conclusion, this large-scale, nationwide retrospective study showed that HBVr in patients with MM was significantly higher, especially among patients who received auto-SCT. Additional prospective studies with long-term observation periods are needed to evaluate the optimal duration of HBV-DNA monitoring and to develop an effective strategy to prevent HBVr in patients with MM.
